# Commentary: How to Count Our Microbes? The Effect of Different Quantitative Microbiome Profiling Approaches

**DOI:** 10.3389/fcimb.2021.627910

**Published:** 2021-03-05

**Authors:** Ching Jian, Anne Salonen, Katri Korpela

**Affiliations:** Human Microbiome Research Program, Faculty of Medicine, University of Helsinki, Helsinki, Finland

**Keywords:** absolute abundance, flow cytometry, quantitative PCR, microbiome, next-generation sequencing, compositional data

The compositional nature of data derived from next-generation sequencing (NGS) is increasingly recognized as one of the cruxes in microbiome research, as relative abundance cannot accurately portray the direction and magnitude of changes of microbial taxa between two experimental conditions or samples (Gloor et al., 2017). Importantly, absolute microbial abundance is on its own an important biological feature (Vandeputte et al., 2017; Contijoch et al., 2019) that cannot be deduced from compositional data using statistical methods. Nevertheless, most NGS-based microbiome studies to date have generally underutilized absolute abundance (Barlow et al., 2020), possibly due to a lack of validation of the available quantitative analysis methods that integrate absolute quantification into a NGS pipeline, termed quantitative microbiome profiling (QMP).

The recent article by Galazzo et al. (2020) compared two popular approaches for QMP, cell-based flow cytometry (Vandeputte et al., 2017), and quantitative PCR (qPCR) applied for DNA extracts (Jian et al., 2020) using a mock mix of bacterial cells and fecal samples. They additionally included a variant of flow cytometry-based QMP, where dead or damaged cells were removed by propidium monoazide (PMA) pre-treatment before sequencing to study how removing extracellular DNA (on average approximately 40% of the total DNA in the fecal samples (Galazzo et al., 2020)), captured in standard NGS but otherwise excluded in flow-cytometry counting, affected the comparisons. The authors found that total bacterial loads quantified by qPCR and flow cytometry were highly correlated when performed in the mock mix. In fecal samples, a lack of commonality in quantitative microbial profiles was found between the cell-based methods and the molecular-based method according to their average Bray-Curtis dissimilarity and average sample rank concordance among abundant bacterial genera. The authors subsequently suggested that flow cytometry is a superior method for QMP over qPCR, mainly based on the finding that the two flow cytometry-based methods showed stronger average sample rank correlations with each other and with relative microbial profiling (RMP) while qPCR generated highly divergent quantitative microbial profiles (Galazzo et al., 2020). We however find this argument unsatisfactory for the reasons described below.

First and foremost, microbial profiles generated by RMP and QMP are expected to differ, as absolute abundance in QMP is influenced by microbial density, such as total bacterial loads that vary vastly between individuals (Contijoch et al., 2019). This expected difference between absolute and relative abundance profiles has been demonstrated in previous studies (Vandeputte et al., 2017) and by Galazzo et al. (2020). If we expected RMP and QMP to be very similar, there would be very little incentive for quantitation. Moreover, there is no gold standard for bacterial enumeration in complex samples. Flow cytometry for bacterial enumeration in complex matrices remains challenging (Frossard et al., 2016) and has not been validated extensively in feces (Barlow et al., 2020). As such, correlation and similarity between the microbial profiles generated by cell-based and molecular-based QMP and by RMP does not constitute a meaningful readout for benchmarking.

Regardless of the accuracy of bacterial quantification in complex samples, cell-based QMP is conceptually problematic. The principal requirement for translating RMP to QMP is to obtain a measure of the total microbiota load *that is being sequenced*, as opposed to the total microbiota load in the original sample. If the microbiota that were sequenced differ from the microbiota that were quantified, the two measures are not comparable, and the QMP result will by default be incorrect. NGS analyzes DNA extracted from live, dead, and damaged cells as well as free DNA (Galazzo et al., 2020). Flow cytometry measures intact cells only, which represent a fraction of the total DNA that is being sequenced, as composition of the live microbiota differs from that of the total DNA in fecal samples (Ben-Amor et al., 2005). On the other hand, many intact cells counted by flow cytometry may not be captured by NGS, depending on DNA extraction protocols, and importantly dead cells and free DNA that form part of RMP, will be missed by flow cytometry. In contrast, qPCR measures exactly the same entity as NGS. Therefore, flow cytometry represents a viable option for enumerating the microbial load only if DNA is extracted from the same entity that is enumerated, namely intact cells. Whether that is desirable, depends on the research question.

There are important biases in NGS that should be considered when designing the quantitation approach, as the same biases need to be present to maximize data comparability. NGS is a multistep procedure of subsampling influenced by some degree of stochasticity as well as various biasing factors in each step, such as DNA extraction efficiency, primer coverage (Jian et al., 2020), and level of PCR inhibitors (Barlow et al., 2020). These factors are shared between NGS and molecular-based QMP (Jian et al., 2020), which is essential for accurate adjustment of RMP to QMP (**Figure 1**). For example, there are significant differences in DNA extraction methods in their efficiency in breaking up the cells of different organisms and extracting the DNA from them, resulting in different taxonomic compositions (Costea et al., 2017). Flow cytometry will be insensitive to all the biases and stochastic variations in NGS and thus will not be an accurate representation of the total microbiota load present in RMP.

**Figure 1:**
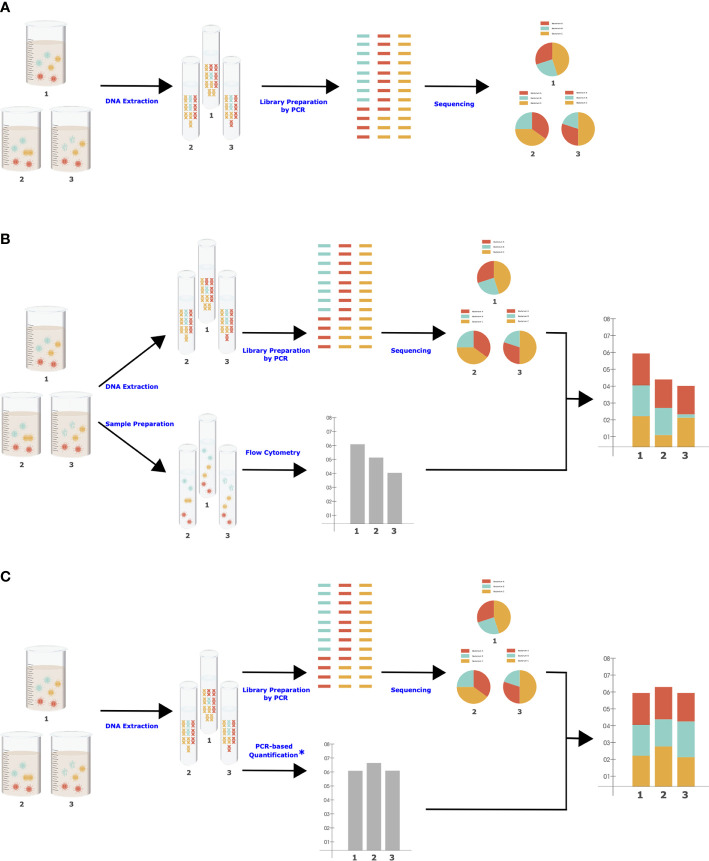
Simplified workflows for NGS **(A)**, cell-based QMP **(B)**, and molecular-based QMP **(C)**. Each step (blue character) can be influenced by various controllable and uncontrollable factors. *Microbial quantification can be performed simultaneously with library preparation in one step (Bogatyrev and Ismagilov, 2020) to eliminate sources of bias.

In conclusion, we praise the comparative study done by Galazzo et al. that demonstrates technical sources of variability arising from different QMP methods. However, we feel the presented results are insufficient to profess that flow cytometry is a preferable method for QMP in feces (Galazzo et al., 2020). Importantly, we call attention to a conceptual pitfall in cell-based QMP that may lead to intractable biases.

## Author Contributions

CJ drafted and revised the manuscript. AS and KK provided feedback and revised the manuscript. All authors contributed to the article and approved the submitted version.

## Conflict of Interest

The authors declare that the research was conducted in the absence of any commercial or financial relationships that could be construed as a potential conflict of interest.

## References

[B1] Barlow J. T. Bogatyrev S. R. Ismagilov R. F. (2020). A quantitative sequencing framework for absolute abundance measurements of mucosal and lumenal microbial communities. Nat. Commun. 11 (1), 2590. 10.1038/s41467-020-16224-6 32444602 PMC7244552

[B2] Ben-Amor K. Heilig H. Smidt H. Vaughan E. E. Abee T. de Vos W. M. (2005). Genetic diversity of viable, injured, and dead fecal bacteria assessed by fluorescence-activated cell sorting and 16S rRNA gene analysis. Appl. Environ. Microbiol. 71 (8), 4679–4689. 10.1128/aem.71.8.4679-4689.2005 16085863 PMC1183343

[B3] Bogatyrev S. R. Ismagilov R. F. (2020). Quantitative microbiome profiling in lumenal and tissue samples with broad coverage and dynamic range via a single-step 16S rRNA gene DNA copy quantification and amplicon barcoding. bioRxiv. 10.1101/2020.01.22.914705v1. [Preprint].

[B4] Contijoch E. J. Britton G. J. Yang C. Mogno I. Li Z. Ng R.. (2019). Gut microbiota density influences host physiology and is shaped by host and microbial factors. Elife 8, e40553. 10.7554/eLife.40553 30666957 PMC6342524

[B5] Costea P. I. Zeller G. Sunagawa S. Pelletier E. Alberti A. Levenez F.. (2017). Towards standards for human fecal sample processing in metagenomic studies. Nat. Biotechnol. 35 (11), 1069–1076. 10.1038/nbt.3960 28967887

[B6] Frossard A. Hammes F. Gessner M. O. (2016). Flow Cytometric Assessment of Bacterial Abundance in Soils, Sediments and Sludge. Front. Microbiol. 7, 903. 10.3389/fmicb.2016.00903 27379043 PMC4905975

[B7] Galazzo G. van Best N. Benedikter B. J. Janssen K. Bervoets L. Driessen C.. (2020). How to Count Our Microbes? The Effect of Different Quantitative Microbiome Profiling Approaches. Front. Cell Infect. Microbiol. 10, 403. 10.3389/fcimb.2020.00403 32850498 PMC7426659

[B8] Gloor G. B. Macklaim J. M. Pawlowsky-Glahn V. Egozcue J. J. (2017). Microbiome Datasets Are Compositional: And This Is Not Optional. Front. Microbiol. 8, 2224. 10.3389/fmicb.2017.02224 29187837 PMC5695134

[B9] Jian C. Luukkonen P. Yki-Järvinen H. Salonen A. Korpela K. (2020). Quantitative PCR provides a simple and accessible method for quantitative microbiota profiling. PLoS One 15 (1), e0227285. 10.1371/journal.pone.0227285 31940382 PMC6961887

[B10] Vandeputte D. Kathagen G. D’Hoe K. Vieira-Silva S. Valles-Colomer M. Sabino J.. (2017). Quantitative microbiome profiling links gut community variation to microbial load. Nature 551 (7681), 507–511. 10.1038/nature24460 29143816

